# Associated diagnostic spectrum and stratified characteristics of patients with eosinophilia: a retrospective study

**DOI:** 10.3389/fmed.2026.1906800

**Published:** 2026-09-11

**Authors:** Dongyu He, Junyi Ke, Lan Yang, Jiaxuan Wu, Jianyue Peng, Lei Li, Weimin Li

**Affiliations:** 1 Department of Pulmonary and Critical Care Medicine, West China Hospital, State Key Laboratory of Respiratory Health and Multimorbidity, Sichuan University, Chengdu, Sichuan, China; 2 Institute of Respiratory Health, Frontiers Science Center for Disease-related Molecular Network, West China Hospital, Sichuan University, Chengdu, Sichuan, China; 3 Precision Medicine Center, Precision Medicine Key Laboratory of Sichuan Province, West China Hospital, Sichuan University, Chengdu, Sichuan, China; 4 The Research Units of West China, Chinese Academy of Medical Sciences, West China Hospital, Chengdu, Sichuan, China; 5 Institute of Respiratory Health and Multimorbidity, West China Hospital, Sichuan University, Chengdu, Sichuan, China

**Keywords:** eosinophilia, associated diagnostic spectrum, retrospective study, eosinophil stratification, solid malignancy

## Abstract

**Background:**

Peripheral blood eosinophilia occurs in diverse clinical conditions, including allergic diseases, infections, autoimmune disorders, hematologic malignancies, and solid malignancies. Although parasitic infection and allergic disease have traditionally been regarded as common settings, the distribution of associated diagnoses may differ in real-world tertiary-care practice. Describing the associated diagnostic spectrum of persistent eosinophilia and its relationship with eosinophil burden and demographics may improve clinical recognition and stratified evaluation.

**Objective:**

To characterize the associated diagnostic spectrum and stratified features of persistent eosinophilia, compare persistent with transient eosinophilia, and provide evidence for standardized clinical evaluation of elevated eosinophil counts.

**Methods:**

This single-center retrospective study used the clinical database of West China Hospital, Sichuan University, including outpatients and inpatients with eosinophilia between 2024 and 2025. Patients with a peak absolute eosinophil count (AEC) > 0.52 × 10^9/L were eligible. Patients with only one AEC measurement or <30 days between their earliest and latest available measurements were excluded. Persistent eosinophilia required at least two AEC values ≥0.50 × 10^9/L separated by ≥30 days; the remaining eligible patients were classified as transient. Baseline demographics and peak AEC were compared between groups. Persistent eosinophilia without a diagnosis generally considered an established explanation for eosinophilia was classified as no established eosinophilia-related diagnosis, and associated diagnostic distributions were assessed across study-specific peak AEC bands, age, sex, and ethnicity. Multivariable analyses were performed.

**Results:**

Of 23,069 patients included, 19,745 had transient and 3,324 had persistent eosinophilia. Patients with persistent eosinophilia were older, more often male, and had higher peak eosinophil counts with greater proportions in the two higher study-specific AEC bands. The mean age difference was 4.03 years (95% CI 3.35–4.71; Hedges g = 0.19). Solid malignancy was the most frequent associated diagnostic category (34.2%), followed by no established eosinophilia-related diagnosis (32.1%). Across the study-specific AEC bands, the proportion without an established related diagnosis decreased, whereas allergic disease increased. Associated diagnostic distribution varied by age and sex, and peak AEC did not differ significantly across solid malignancy subtypes. Among patients without an established related diagnosis, CKD was recorded in 565/1,067 (53.0%); adjusted CKD associations were weak and exploratory.

**Conclusions:**

In this tertiary-care cohort, persistent eosinophilia occurred across a broad associated diagnostic spectrum, with solid malignancy and no established eosinophilia-related diagnosis as the two most frequent categories. These descriptive findings do not establish causation. Differential repeat testing, referral selection, unmeasured medication exposure, and residual confounding limit clinical interpretation.

## 1 Introduction

Eosinophils are involved in tissue homeostasis, host defense, and immune regulation, whereas inappropriate eosinophil activation can contribute to tissue injury across multiple organ systems (1). Because of the complexity and heterogeneity of eosinophil-associated conditions, evaluating peripheral blood eosinophilia remains a common clinical challenge. Persistent eosinophilia may warrant particular attention, but persistence alone does not identify its cause.

Previous studies have generally identified atopic diseases and parasitic infections as common settings for eosinophilia (2). This view, however, does not fully align with our recent clinical experience. In routine practice, the distribution of associated diagnoses of eosinophilia may vary substantially according to patient demographics, environmental exposures, and the case mix of the treating institution.

Missed diagnoses, delayed recognition, and inconsistent management remain frequent in patients with eosinophilia. Mild eosinophilia is especially prone to being overlooked, resulting in insufficient specialist evaluation and missed opportunities for early targeted treatment (3, 4). Therefore, a contemporary understanding of the associated diagnostic distribution of eosinophilia in real-world practice is needed.

We used a large clinical database from West China Hospital, Sichuan University, to include patients with eosinophilia seen across outpatient and inpatient settings over a 2-year period. We systematically examined the associated diagnostic spectrum of persistent eosinophilia to inform a stepwise diagnostic investigation and provide epidemiologic evidence for standardized, stratified management.

## 2 Materials and methods

### 2.1 Study design and data source

This was a single-center retrospective observational study based on the hospital information system and related clinical databases of West China Hospital, Sichuan University. The study population comprised patients with at least one record of elevated peripheral blood eosinophils during outpatient or inpatient care between January 1, 2024, and December 31, 2025. Because peripheral blood eosinophilia can occur in allergic diseases, infections, autoimmune diseases, solid malignancies, hematologic disorders, and many other clinical conditions, a real-world database from a large tertiary hospital provides an appropriate setting for an associated diagnostic spectrum study.

### 2.2 Study population

#### 2.2.1 Inclusion criteria

Patients were eligible if they had at least one absolute eosinophil count (AEC) > 0.52 × 10^9/L during the study period. This threshold corresponded to the upper reference limit for eosinophils in the Chinese health-industry standard WS/T 405-2012 (5).

Patients with only one AEC measurement or <30 days between their earliest and latest available measurements were excluded. Persistent eosinophilia was defined as at least two AEC values ≥0.50 × 10^9/L separated by ≥30 days. Among patients with at least two AEC measurements spanning ≥30 days, those who did not meet the persistence criterion were classified as having transient eosinophilia. Persistence was assessed using all available dated AEC measurements. The cohort screening process and classification criteria are summarized in Supplementary Table 7.

#### 2.2.2 Exclusion criteria

Patients were excluded if they had missing key demographic information or no clinical diagnostic record available for review. Patients with a recorded diagnosis that was not an established explanation for eosinophilia remained eligible in the no established eosinophilia-related diagnosis category.

### 2.3 Variables and definitions

#### 2.3.1 Baseline characteristics

Age, sex, ethnicity, and other demographic variables were collected. The highest eosinophil count recorded during the study period was used as the primary measure of eosinophil burden. Dates of AEC testing and inpatient or outpatient contact were also extracted when available. No reliable structured medication-exposure table was available; medication exposure was therefore not modeled.

#### 2.3.2 Study-specific peak AEC bands

For descriptive analyses, patients with persistent eosinophilia were grouped by peak AEC into three study-specific analytical bands: >0.52 to <1.00, 1.00 to <1.50, and ≥1.50 × 10^9/L. Comparable cutoffs (0.50–0.99, 1.00–1.49, and ≥1.50 × 10^9/L) have been used in previous retrospective eosinophilia cohorts (6, 7), but these study-specific bands are not conventional severity grades. Commonly used count-based grades define mild eosinophilia as 0.50 to <1.50 × 10^9/L, moderate eosinophilia as 1.50 to 5.00 × 10^9/L, and severe eosinophilia as >5.00 × 10^9/L; current consensus defines hypereosinophilia at ≥1.50 × 10^9/L (8–10). In the persistent cohort, 2,792 patients had a peak AEC below 1.50 × 10^9/L and only 36 exceeded 5.00 × 10^9/L. We retained the originally specified study bands to preserve analytical resolution below 1.50 × 10^9/L while retaining the consensus hypereosinophilia threshold.

#### 2.3.3 Associated diagnostic classification

Based on discharge diagnoses, outpatient diagnoses, specialist consultation records, and relevant investigations, patients with persistent eosinophilia were assigned to one mutually exclusive associated diagnostic category: (1) solid malignancy; (2) infectious disease; (3) allergic disease; (4) autoimmune disease; (5) hematologic malignancy; (6) other diagnoses, including chronic obstructive pulmonary disease and acute or chronic graft-versus-host disease; and (7) no established eosinophilia-related diagnosis. This classification was informed by previous studies and reviews (8, 9, 11).

For this study, the solid malignancy category included malignant solid neoplasms for which pathology or biopsy confirmation performed at our hospital or another hospital was documented in the medical record; benign tumors were excluded. When autoimmune disease coexisted with another potentially relevant diagnosis, autoimmune disease was prioritized. For remaining overlaps, the single category retained in the final chart-review dataset was used. The available retrospective records did not support standardized prospective causal adjudication; these categories therefore describe associated diagnoses rather than established causes of eosinophilia.

In this study, no established eosinophilia-related diagnosis did not mean that the patient had no clinical diagnosis. Rather, it indicated that the recorded diagnosis was not generally considered an established explanation for eosinophilia in current clinical practice or research. For these patients, recorded diagnoses were summarized descriptively without assigning a causal relationship to eosinophilia.

#### 2.3.4 Solid malignancy subtypes

Solid malignancies were further classified by system of origin into digestive system, respiratory system, urinary system, breast, head and neck, bone and soft tissue, reproductive system, endocrine system, skin, nervous system, eye and oral malignancies, unclassified solid malignancies, and other solid malignancy subtypes. Unclassified malignancies included multiple-site malignancies, non-cutaneous melanoma, Kaposi sarcoma, and malignancies that could not be reliably assigned to a single system. In analyses of study-specific peak AEC bands, malignancy subtypes with 20 or fewer cases were combined into the other solid malignancies category. This subtype classification was descriptive and did not establish that malignancy caused eosinophilia.

### 2.4 Statistical analysis

Continuous variables were summarized as mean ± standard deviation or median with interquartile range, as appropriate. Categorical variables were summarized as counts and percentages. Age was compared using Welch's *t* test because variances differed; the mean difference, 95% confidence interval, and Hedges g were reported. Peak AEC was compared using the Wilcoxon rank-sum test, and proportions using the chi-square test. Cochran-Armitage trend tests were used for associated diagnostic distributions across ordered study-specific AEC bands and age groups; sex and ethnicity comparisons used Pearson chi-square tests with Yates continuity correction. These diagnosis-specific *P* values were Bonferroni-corrected for seven categories. For solid malignancy subtype analyses, subtypes with 20 or fewer cases were combined into an other category. Differences in peak AEC across subtypes were assessed using the Kruskal–Wallis test, and differences in study-specific AEC band distributions using a fixed-margin Monte Carlo Pearson chi-square test with 1,000,000 replicates. A two-sided Jonckheere–Terpstra test assessed the unadjusted ordered trend in peak AEC across CKD stages. A separate multivariable logistic model examined recorded CKD status among patients without an established related diagnosis using age, sex, ethnicity, and log2-transformed peak AEC. CKD-stage models among patients with CKD were adjusted for age, sex, and ethnicity. All statistical tests were two-sided, and *P* < 0.05 was considered statistically significant.

Multivariable logistic regression modeled persistent versus transient classification with heteroscedasticity-consistent (HC3) standard errors. Model 1 included age per 10 years, sex, and ethnicity; Model 2 additionally included log2-transformed peak AEC. Both models included all 23,069 patients who met the cohort eligibility and repeat-measurement criteria.

### 2.5 Ethics statement

This retrospective study was based on existing clinical data and was approved by the Ethics Committee of West China Hospital, Sichuan University, with a waiver of informed consent. Ethics approval number: 2026-[223].

## 3 Results

### 3.1 Baseline comparison between transient and persistent eosinophilia

**Figure 1:**
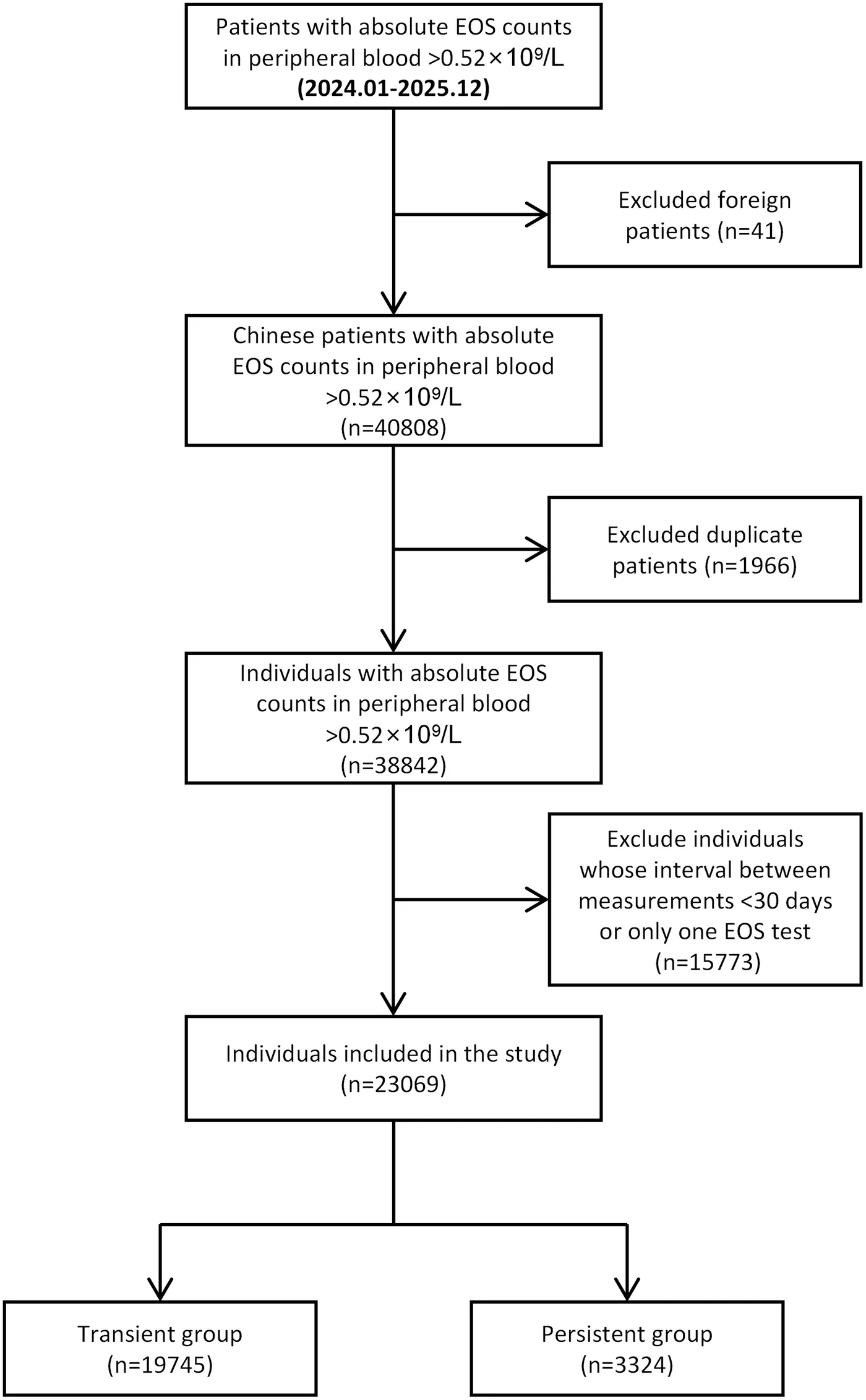
Study flowchart. Of 38,842 candidates, 15,773 were excluded because they had only one AEC measurement or <30 days between their earliest and latest available measurements. The final cohort included 19,745 patients with transient and 3,324 with persistent eosinophilia.

**Figure 2:**
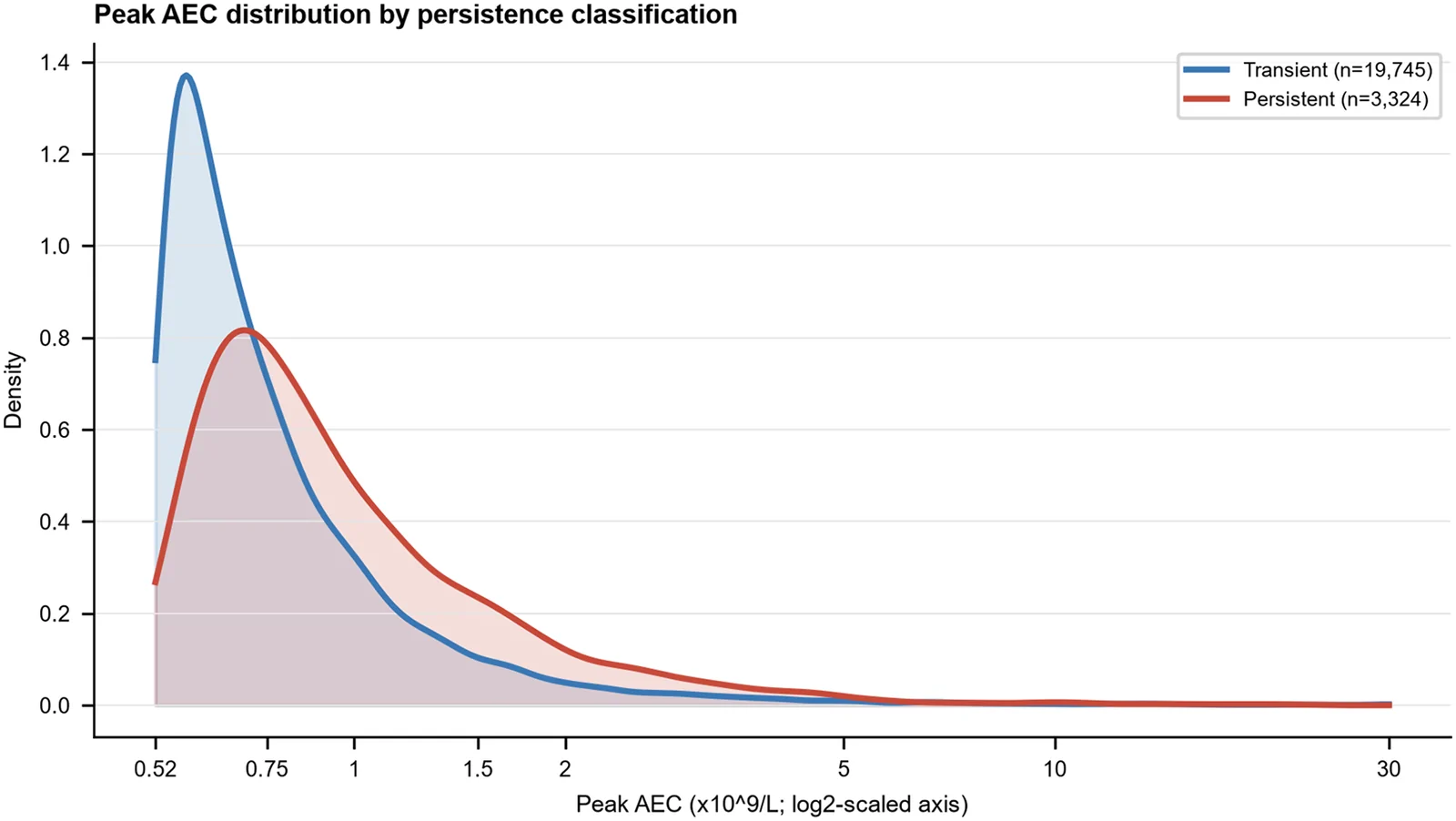
Distribution of peak eosinophil counts in patients with transient and persistent eosinophilia.

A total of 23,069 patients with eosinophilia were included: 19,745 with transient eosinophilia and 3,324 with persistent eosinophilia (Figure 1). Baseline characteristics and peak eosinophil counts are summarized below. Mean age was 50.27 ± 21.56 years in the transient group and 54.30 ± 18.05 years in the persistent group; the mean difference was 4.03 years (95% CI 3.35–4.71; *P* < 0.001), with a small standardized effect (Hedges g = 0.19). The persistent group was also more often male and of Han ethnicity, and its age distribution included fewer minors and more middle-aged or older patients. The persistent group had higher peak AEC (*P* < 0.001) and larger proportions in the 1.00 to <1.50 and ≥1.50 × 10^9/L study-specific bands (Figure 2, Table 1, Supplementary Figure 1A).

**Table 1:** Baseline characteristics of patients with transient and persistent eosinophilia. The analytical peak AEC bands were study-specific and were not labeled as conventional severity grades.

Characteristics	Level	Transient	Persistent	*P* value
Numbers		19,745	3,324	
Age (years, mean ± SD)		50.27 ± 21.56	54.30 ± 18.05	<0.001
Sex (%)	Male	12,409 (62.8%)	2,251 (67.7%)	<0.001
	Female	7,336 (37.2%)	1,073 (32.3%)	
Ethnicity (%)	Han	17,891 (90.6%)	3,117 (93.8%)	<0.001
	Minority	1,854 (9.4%)	207 (6.2%)	
Peak AEC (median [IQR], x10^9/L)		0.68 [0.58, 0.88]	0.84 [0.68, 1.18]	<0.001
Study-specific peak AEC band (%)	>0.52 to <1.00	16,146 (81.8%)	2,132 (64.1%)	<0.001
	1.00 to <1.50	2,203 (11.2%)	660 (19.9%)	
	≥1.50	1,396 (7.1%)	532 (16.0%)	
Age group (years, %)	<18	2,144 (10.9%)	131 (3.9%)	<0.001
	[18–40)	3,653 (18.5%)	585 (17.6%)	
	[40–60)	6,571 (33.3%)	1,248 (37.5%)	
	[60–75)	5,416 (27.4%)	1,026 (30.9%)	
	≥75	1,961 (9.9%)	334 (10.0%)	

To determine whether these differences persisted across demographic subgroups, we performed subgroup analyses by age (<18, [18–40), [40–60), [60–75), and ≥75 years), sex (male/female), and ethnicity (Han/minority). Across all 9 subgroups, peak eosinophil counts were higher in the persistent group than in the transient group (Supplementary Figure 1B). These unadjusted subgroup comparisons do not exclude confounding.

In Model 2, age per 10 years (aOR 1.09, 95% CI 1.07–1.10), male sex (aOR 1.21, 95% CI 1.12–1.31), Han ethnicity (aOR 1.53, 95% CI 1.31–1.78), and each doubling of peak AEC (aOR 1.75, 95% CI 1.67–1.84) were associated with persistent classification (Supplementary Table 6).

### 3.2 Overall associated diagnostic spectrum and trends across study-specific AEC bands

**Figure 3:**
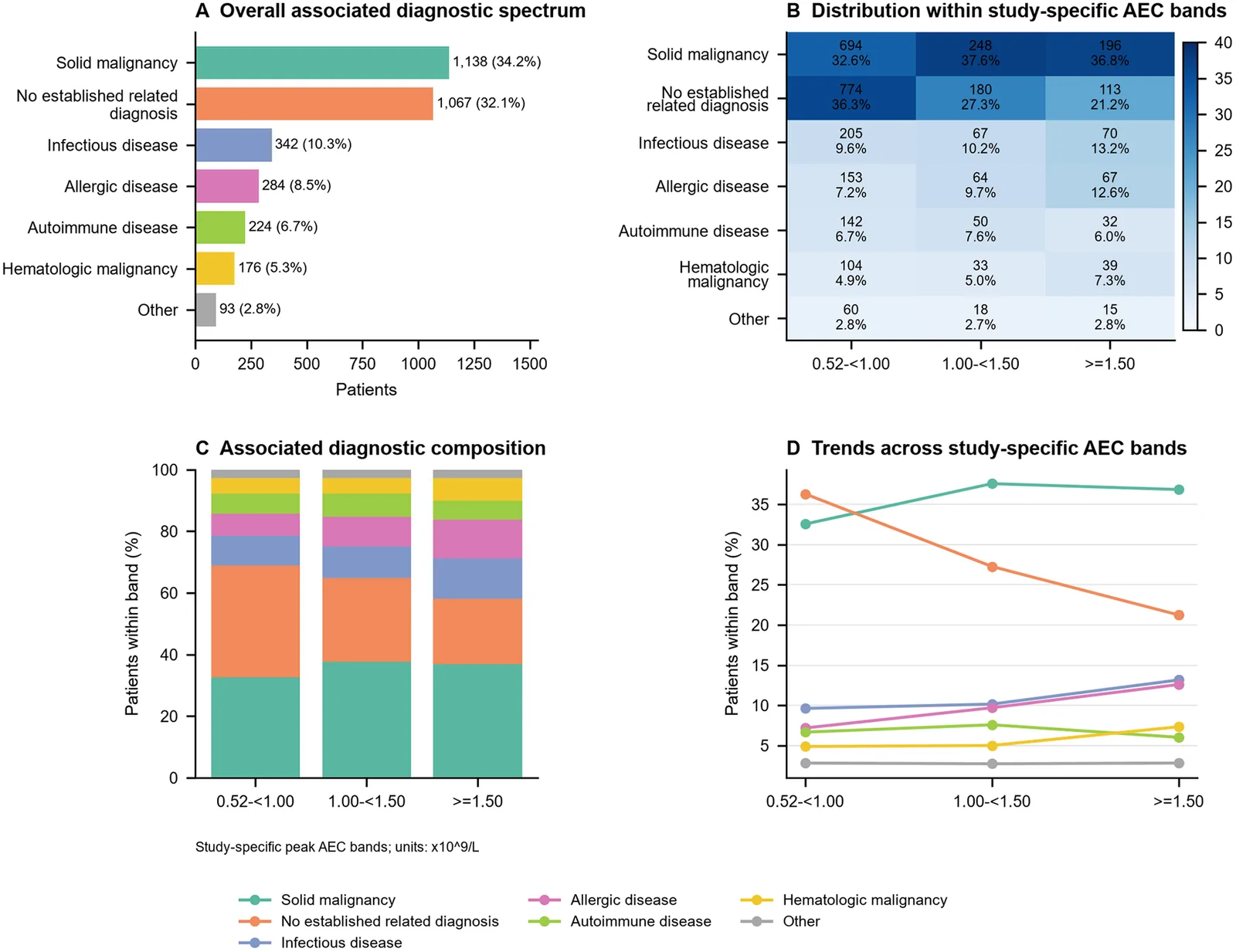
Associated diagnostic distributions across study-specific peak AEC bands among patients with persistent eosinophilia. **(A)** Overall associated diagnostic spectrum. **(B)** Heatmap. **(C)** Stacked bar chart. **(D)** Trend analysis.

Among the 3,324 patients with persistent eosinophilia, solid malignancy and no established eosinophilia-related diagnosis were the two most frequent mutually exclusive associated diagnostic categories, accounting for 34.2% and 32.1%, respectively (Figure 3A). We then examined their distributions across the study-specific peak AEC bands. The proportion without an established related diagnosis decreased significantly as the AEC band increased (*P* < 0.001), whereas the proportion with allergic disease increased significantly (*P* < 0.001). No established related diagnosis was the most frequent category in the lowest band, whereas solid malignancy was the most frequent associated diagnosis in the two higher bands. Infectious disease and hematologic malignancy increased numerically but did not remain significant after Bonferroni correction. Autoimmune disease and other diagnoses showed no clear trend (Figures 3B–D, Supplementary Table 1).

### 3.3 Associations between demographic features and associated diagnoses

We further examined associations between baseline demographic features and associated diagnostic distribution among patients with persistent eosinophilia. In sex-stratified analyses, autoimmune diseases were significantly more common in women than in men (14.1% vs. 3.2%, *P* < 0.001), whereas solid malignancy (30.8% vs. 35.9%, *P* = 0.035) and no established eosinophilia-related diagnosis (28.8% vs. 33.7%, *P* = 0.039) were more common in men. Infectious diseases, allergic diseases, hematologic malignancies, and other diagnoses did not differ significantly by sex (Supplementary Figure 2, Supplementary Table 2). These comparisons were unadjusted for other clinical factors.

**Figure 4:**
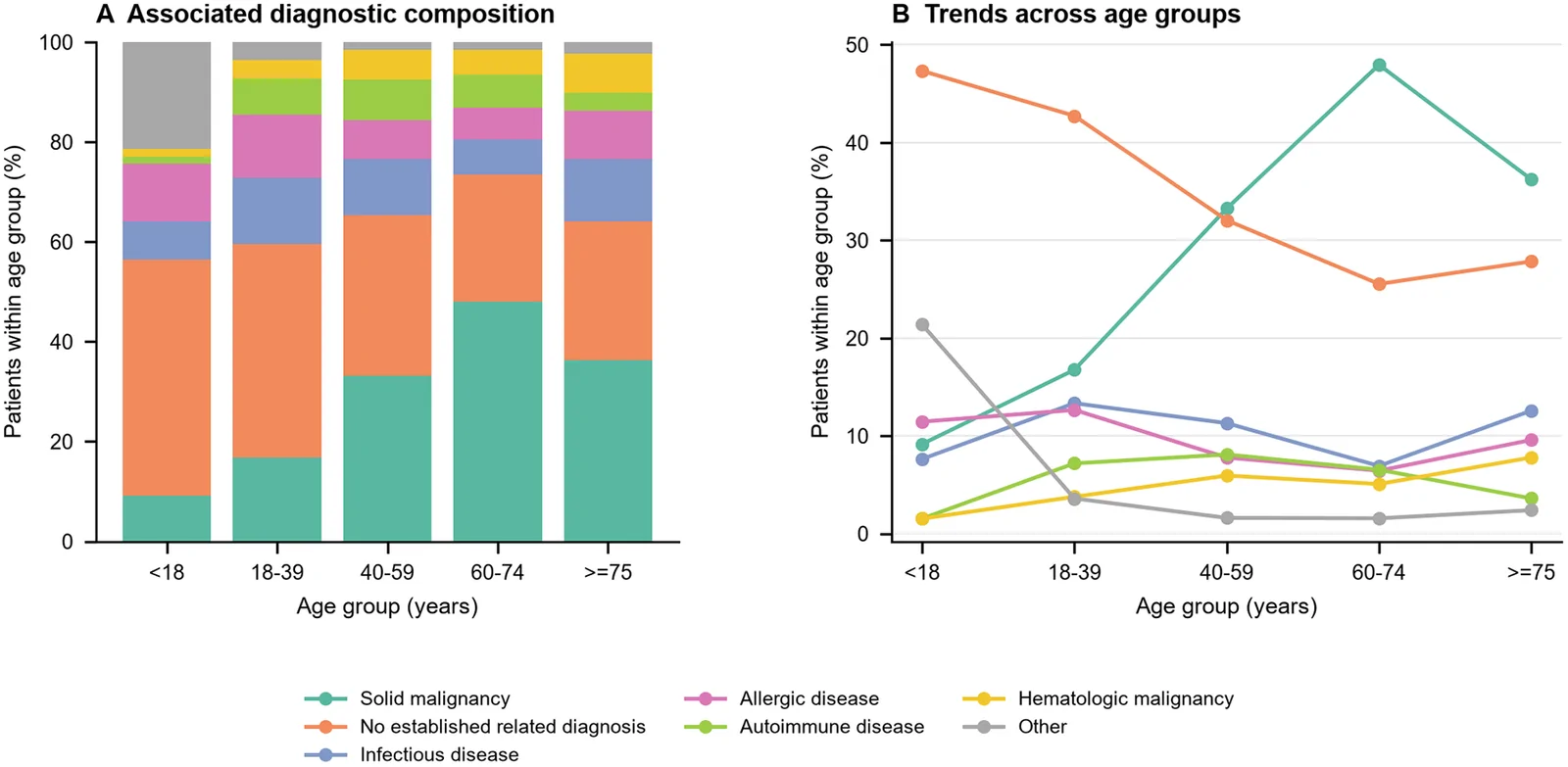
Associated diagnostic distributions across age groups among patients with persistent eosinophilia. Left: stacked bar chart. Right: trend analysis.

By ethnicity, no associated diagnostic category differed significantly between Han and minority patients after correction (Supplementary Figure 3, Supplementary Table 3). By age group, the distributions of solid malignancy, no established eosinophilia-related diagnosis, allergic disease, and other diagnoses differed significantly (*P* < 0.05). Solid malignancy was most frequent in middle-aged and older patients, whereas no established related diagnosis and allergic disease were most frequent in middle-aged patients. The proportion of other diagnoses declined with age (Figure 4, Supplementary Table 4), mainly because this category included graft-versus-host disease in younger patients. These patterns may reflect referral and diagnostic intensity and should not be interpreted as age-specific causation.

### 3.4 Solid malignancy subtypes and eosinophil burden

Among 1,138 patients with solid malignancy, the most common malignancy subtypes were digestive system malignancies (37.9%), respiratory system malignancies (30.1%), urinary system malignancies (13.4%), and breast malignancies (8.3%); together, these four groups accounted for more than 80% of all solid malignancies. Less frequent subtypes were combined into other solid malignancies for statistical analysis because of their small sample sizes (Table 2).

**Table 2:** Baseline characteristics, peak AEC, and study-specific peak AEC bands by solid malignancy subtype.

Solid malignancy subtype	Numbers (%)	Peak AEC (median [IQR], x10^9/L)	>0.52 to <1.00 (n, %)	1.00 to <1.50 (n, %)	≥1.50 (n, %)	Age (mean ± SD)	Male (%)
Digestive system	431 (37.9%)	0.86 (0.69–1.26)	252 (58.5%)	108 (25.1%)	71 (16.5%)	59.9 ± 12.2	81.2%
Respiratory system	343 (30.1%)	0.87 (0.70–1.25)	212 (61.8%)	76 (22.2%)	55 (16.0%)	62.4 ± 12.6	74.1%
Urinary system	153 (13.4%)	0.91 (0.69–1.41)	93 (60.8%)	26 (17.0%)	34 (22.2%)	62.8 ± 13.2	85.6%
Breast	94 (8.3%)	0.81 (0.65–1.10)	67 (71.3%)	15 (16.0%)	12 (12.8%)	55.1 ± 12.5	1.1%
Head & neck	26 (2.3%)	0.78 (0.66–1.22)	18 (69.2%)	4 (15.4%)	4 (15.4%)	57.6 ± 15.1	92.3%
*Other solid malignancies*	**91** (**8.0%)**	**0.90** (**0.73–1.40)**	**52** (**57.1%)**	**19** (**20.9%)**	**20** (**22.0%)**	**49.4 ± 20.7**	**51**.**6%**
Bone & soft tissue	20 (1.8%)	0.83 (0.78–1.33)	12 (60.0%)	5 (25.0%)	3 (15.0%)	46.3 ± 23.5	55.0%
Reproductive system	18 (1.6%)	0.95 (0.75–1.77)	10 (55.6%)	3 (16.7%)	5 (27.8%)	56.9 ± 14.9	16.7%
Endocrine system	16 (1.4%)	0.90 (0.76–1.52)	10 (62.5%)	2 (12.5%)	4 (25.0%)	52.5 ± 14.0	37.5%
Skin	11 (1.0%)	1.48 (0.73–2.02)	4 (36.4%)	2 (18.2%)	5 (45.5%)	51.7 ± 15.5	54.5%
Eye and oral tumors	10 (0.9%)	0.99 (0.77–1.15)	5 (50.0%)	3 (30.0%)	2 (20.0%)	55.6 ± 11.2	80.0%
Nervous system	9 (0.8%)	0.69 (0.65–0.87)	7 (77.8%)	1 (11.1%)	1 (11.1%)	48.5 ± 23.4	77.8%
Unclassified solid malignancies	7 (0.6%)	0.82 (0.76–1.07)	4 (57.1%)	3 (42.9%)	0 (0.0%)	20.0 ± 30.1	85.7%

Bold values indicate the aggregate “Other solid malignancies” category used in the statistical analysis; the indented rows show its component subtypes and are not additional patients.

Across the 6 analytic solid malignancy subtype groups, peak AEC did not differ significantly (*P* = 0.199, Supplementary Figure 4). Urinary system malignancies had the highest median peak AEC (0.91), whereas head and neck malignancies had the lowest median value (0.78). Study-specific peak AEC band distributions also did not differ significantly across malignancy subtypes (Monte Carlo *P* = 0.205, Supplementary Figure 5). Urinary system malignancies accounted for a higher proportion of the ≥1.50 × 10^9/L band (22.2%), whereas head and neck malignancies were more often observed in the lowest study-specific band (69.2%). Given the imbalance in sample size across subtypes, these findings require validation in larger cohorts.

### 3.5 Recorded diagnoses in patients without an established eosinophilia-related diagnosis

**Figure 5:**
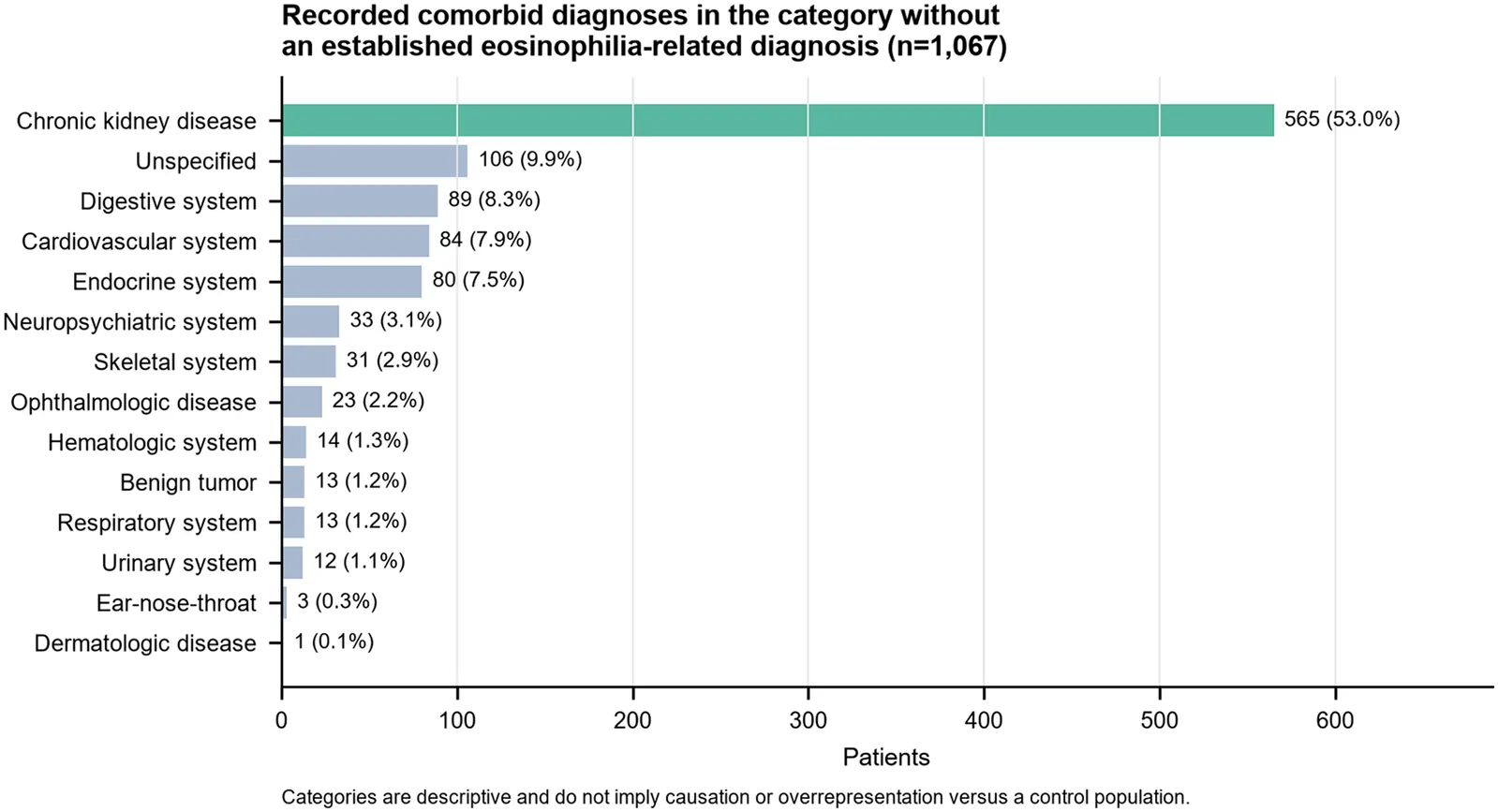
Recorded diagnoses among patients without an established eosinophilia-related diagnosis. Categories are descriptive and do not imply causation or overrepresentation relative to a control population.

Among 1,067 patients with persistent eosinophilia and no established eosinophilia-related diagnosis, chronic kidney disease (CKD) was the most frequently recorded diagnosis, accounting for 565 (53.0%). Other recorded conditions included digestive system diseases, cardiovascular diseases, endocrine system diseases, and neuropsychiatric disorders. The unspecified category included 106 patients (9.9%) with health-examination findings, allogeneic organ transplantation, trauma or burns, obesity, or other conditions that could not be classified more precisely (Figure 5). These percentages describe this selected category and were not compared with a general or hospital control population.

Compared with patients without CKD, those with CKD were older, whereas peak AEC, study-specific AEC bands, sex, and ethnicity did not differ significantly (Supplementary Table 5). In the multivariable CKD-status model among 1,067 patients without an established related diagnosis, age per 10 years was associated with recorded CKD (aOR 1.19, 95% CI 1.12–1.27; *P* < 0.001), whereas male sex (aOR 1.27, 95% CI 0.97–1.66; *P* = 0.084) and Han ethnicity (aOR 1.35, 95% CI 0.79–2.29; *P* = 0.273) were not statistically significant; each doubling of peak AEC had an aOR of 0.82 (95% CI 0.67–0.99; *P* = 0.041). Among 565 patients with CKD, the unadjusted two-sided Jonckheere–Terpstra trend test gave *P* = 0.011. Each one-stage increase in CKD stage was associated with a peak AEC ratio of 1.032 (95% CI 1.005–1.060; *P* = 0.020) after adjustment for age, sex, and ethnicity. The adjusted odds ratio for the ≥1.50 × 10^9/L band was 1.26 (95% CI 0.98–1.62; *P* = 0.069). These weak and partly nonsignificant associations do not demonstrate that CKD caused eosinophilia (Supplementary Figure 6, Supplementary Table 8A,B).

## 4 Discussion

In this retrospective study, we evaluated the associated diagnostic spectrum of 3,324 patients with persistent peripheral blood eosinophilia and compared them with 19,745 patients classified as transient. First, peak AEC was higher in patients with persistent eosinophilia across demographic subgroups, and the two higher study-specific AEC bands were more frequent. Second, solid malignancy was the most frequent associated diagnosis, but was not established as a cause of eosinophilia. Third, the proportion of allergic disease increased across the study-specific AEC bands. Fourth, the associated diagnostic spectrum varied with age. Fifth, CKD was the most frequently recorded diagnosis among patients without an established related diagnosis, but adjusted CKD associations were weak and exploratory.

In the primary cohort, age, male sex, Han ethnicity, and peak AEC were associated with persistent classification after adjustment. Although all patients met the minimum repeat-measurement criterion, testing frequency and follow-up duration were not standardized. Differences in surveillance intensity may therefore have influenced the probability of meeting the persistence criterion.

Diagnostic and outcome data were unavailable for the transient group. Consequently, transient classification should not be interpreted as the natural history of isolated eosinophilia or as evidence that deferred evaluation is safe. Future prospective studies should characterize associated diagnoses and clinical outcomes in patients with transient eosinophilia.

In this large tertiary hospital cohort, solid malignancy was the most frequent associated diagnosis among patients with persistent eosinophilia (1,138/3,324, 34.2%). The retrospective design, absence of standardized causal adjudication, and lack of temporal and structured medication-exposure data do not support the inference that malignancy caused or drove persistent eosinophilia.

These findings should not be converted directly into screening recommendations. Empirical antiparasitic or antiallergic treatment would not address most recorded associated diagnoses in this setting, but the categories were descriptive and do not establish which condition caused eosinophilia. Clinical evaluation should remain individualized according to symptoms, AEC magnitude and trajectory, exposures, treatments, and established guidelines.

Among solid malignancies, digestive, respiratory, and urinary system malignancies accounted for 81.4% of cases. Cytokine signaling and treatment exposure are biologically plausible hypotheses (12–15), and antitumor treatments may produce drug-related eosinophilia. In the present analysis, however, peak AEC did not differ significantly across analytic subtype groups. The available data lacked temporal linkage and structured medication exposure and therefore do not establish a malignancy-specific mechanism or causal relationship.

Age-stratified analyses showed that the proportion with solid malignancy increased from 9.2% (12/131) in patients younger than 18 years to 48.0% (492/1,026) in those aged 60–74 years, making it the most frequent associated category in that age group. The proportion without an established related diagnosis decreased with age, which may reflect referral and investigation practices. The female predominance of autoimmune disease was consistent with established epidemiology (16). These patterns should not be used to infer that a demographic characteristic identifies the cause of eosinophilia.

In this study, 32.1% of patients with persistent eosinophilia had no diagnosis considered an established explanation for eosinophilia. This proportion was highest in the lowest study-specific AEC band and decreased to 21.2% in the highest band. Differences in clinical attention and diagnostic workup are one possible explanation. Less marked eosinophilia may receive less extensive evaluation, whereas higher AEC may trigger investigation for allergic, infectious, malignant, hematologic, and autoimmune diagnoses. However, diagnostic workup intensity was not measured sufficiently to test this hypothesis.

Among patients without an established eosinophilia-related diagnosis, CKD was recorded in 53.0%. Because there was no appropriate control population, this percentage cannot be interpreted as overrepresentation. In the adjusted CKD-status model, older age was positively associated with recorded CKD, while peak AEC showed a small inverse association; sex and ethnicity were not statistically significant. Among patients with CKD, the adjusted association between CKD stage and peak AEC was small, and the adjusted ≥1.50 × 10^9/L-band model was not statistically significant. Mechanistic pathways discussed in prior studies (17–22) remain hypotheses because they were not measured here. Studies have examined eosinophil-related findings in parasitic disease and cancer (23, 24), and age-related changes in eosinophil function and eosinophilic disorders have also been reported (25, 26). Guidance on hematologic genetic testing and retrospective CKD cohorts further informs the evaluation of selected patients (27–29), although these sources do not establish causal relationships in our cohort.

This study has limitations. First, it was a single-center retrospective study in a tertiary referral hospital, limiting generalizability to community and primary-care populations. Second, testing frequency and follow-up duration were not standardized, and detailed longitudinal test histories beyond the minimum repeat-measurement requirement were unavailable for analysis; differential surveillance and some classification error therefore remain possible. Third, associated diagnoses were assigned from records without uniform prospective causal adjudication; the mutually exclusive rule reduced double counting but could not exclude every competing explanation. Fourth, diagnostic data were unavailable for the transient group. Fifth, structured medication-exposure data were unavailable, so drug-related eosinophilia and treatment effects could not be adjusted for. Sixth, measured covariate adjustment cannot remove unmeasured confounding. Finally, the CKD analyses lacked an external control population and should be considered exploratory.

In conclusion, in this large Chinese tertiary hospital cohort, persistent eosinophilia occurred across a broad associated diagnostic spectrum. Solid malignancy was the most frequent associated diagnosis, followed by no established eosinophilia-related diagnosis. These descriptive findings do not establish causality. Prospective multicenter studies with standardized repeat testing, medication ascertainment, appropriate comparison populations, and diagnostic adjudication are needed.

## Data Availability

The data supporting the findings of this study are available from the corresponding author (Weimin Li, MD, PhD) on reasonable request. Requests will be considered upon submission of a peer-reviewed research proposal, institutional ethics approval from the requesting institution, and a signed data-access agreement.
