# Supplementary material for: Associated diagnostic spectrum and stratified characteristics of patients with eosinophilia: a retrospective study
Source: Front Med (Lausanne). 2026 Sep 11;13:1906800. doi: 10.3389/fmed.2026.1906800 (PMC13612347; doi:10.3389/fmed.2026.1906800)
Supplement: Supplementary file 1 [file Table1.docx]

**Supplementary Materials**

**Supplementary Figure 1. Peak AEC bands and subgroup comparisons in patients classified as having transient or persistent eosinophilia. A. Study-specific AEC bands. B. Subgroup comparisons of peak AEC.**

**Supplementary Figure 2. Associated diagnostic categories by sex among patients with persistent eosinophilia.**

**Supplementary Figure 3. Associated diagnostic categories by ethnicity among patients with persistent eosinophilia.**

**Supplementary Figure 4. Peak AEC by solid malignancy subtype. Subtypes with 20 or fewer cases were combined as Other.**

**Supplementary Figure 5. Study-specific peak AEC bands by solid malignancy subtype.**

**Supplementary Figure 6. Peak AEC by CKD stage and primary kidney disease.**

**Supplementary Table 1. Associated diagnostic categories across study-specific peak AEC bands among patients with persistent eosinophilia.**

| **Associated diagnostic category** | **>0.52 to <1.00** | **1.00 to <1.50** | **>=1.50** | **Bonferroni P** |
| --- | --- | --- | --- | --- |
| Solid malignancy (n, %) | 694 (32.6%) | 248 (37.6%) | 196 (36.8%) | 0.112 |
| No established related diagnosis (n, %) | 774 (36.3%) | 180 (27.3%) | 113 (21.2%) | <0.001 |
| Infectious disease (n, %) | 205 (9.6%) | 67 (10.2%) | 70 (13.2%) | 0.175 |
| Allergic disease (n, %) | 153 (7.2%) | 64 (9.7%) | 67 (12.6%) | <0.001 |
| Autoimmune disease (n, %) | 142 (6.7%) | 50 (7.6%) | 32 (6.0%) | 1 |
| Hematologic malignancy (n, %) | 104 (4.9%) | 33 (5.0%) | 39 (7.3%) | 0.301 |
| Other diagnosis (n, %) | 60 (2.8%) | 18 (2.7%) | 15 (2.8%) | 1 |

**Supplementary Table 2. Associated diagnostic categories by sex among patients with persistent eosinophilia.**

| **Associated diagnostic category** | **Male** | **Female** | **Bonferroni P** |
| --- | --- | --- | --- |
| Solid malignancy (n, %) | 807 (35.9%) | 331 (30.8%) | 0.035 |
| No established related diagnosis (n, %) | 758 (33.7%) | 309 (28.8%) | 0.039 |
| Infectious disease (n, %) | 247 (11.0%) | 95 (8.9%) | 0.482 |
| Allergic disease (n, %) | 186 (8.3%) | 98 (9.1%) | 1 |
| Autoimmune disease (n, %) | 73 (3.2%) | 151 (14.1%) | <0.001 |
| Hematologic malignancy (n, %) | 113 (5.0%) | 63 (5.9%) | 1 |
| Other diagnosis (n, %) | 67 (3.0%) | 26 (2.4%) | 1 |

**Supplementary Table 3. Associated diagnostic categories by ethnicity among patients with persistent eosinophilia.**

| **Associated diagnostic category** | **Han** | **Minority** | **Bonferroni P** |
| --- | --- | --- | --- |
| Solid malignancy (n, %) | 1,078 (34.6%) | 60 (29.0%) | 0.818 |
| No established related diagnosis (n, %) | 1,002 (32.1%) | 65 (31.4%) | 1 |
| Infectious disease (n, %) | 310 (9.9%) | 32 (15.5%) | 0.112 |
| Allergic disease (n, %) | 269 (8.6%) | 15 (7.2%) | 1 |
| Autoimmune disease (n, %) | 207 (6.6%) | 17 (8.2%) | 1 |
| Hematologic malignancy (n, %) | 164 (5.3%) | 12 (5.8%) | 1 |
| Other diagnosis (n, %) | 87 (2.8%) | 6 (2.9%) | 1 |

**Supplementary Table 4. Associated diagnostic categories by age group among patients with persistent eosinophilia.**

| **Associated diagnostic category** | **<18** | **[18, 40)** | **[40,60)** | **[60, 75)** | **>=75** | **Bonferroni P** |
| --- | --- | --- | --- | --- | --- | --- |
| Solid malignancy (n, %) | 12 (9.2%) | 98 (16.8%) | 415 (33.3%) | 492 (48.0%) | 121 (36.2%) | <0.001 |
| No established related diagnosis (n, %) | 62 (47.3%) | 250 (42.7%) | 400 (32.1%) | 262 (25.5%) | 93 (27.8%) | <0.001 |
| Infectious disease (n, %) | 10 (7.6%) | 78 (13.3%) | 141 (11.3%) | 71 (6.9%) | 42 (12.6%) | 0.574 |
| Allergic disease (n, %) | 15 (11.5%) | 74 (12.6%) | 97 (7.8%) | 66 (6.4%) | 32 (9.6%) | 0.026 |
| Autoimmune disease (n, %) | 2 (1.5%) | 42 (7.2%) | 101 (8.1%) | 67 (6.5%) | 12 (3.6%) | 1 |
| Hematologic malignancy (n, %) | 2 (1.5%) | 22 (3.8%) | 74 (5.9%) | 52 (5.1%) | 26 (7.8%) | 0.066 |
| Other diagnosis (n, %) | 28 (21.4%) | 21 (3.6%) | 20 (1.6%) | 16 (1.6%) | 8 (2.4%) | <0.001 |

**Supplementary Table 5. Characteristics of patients with and without CKD in the category without an established eosinophilia-related diagnosis.**

| **Characteristics** | **CKD** | **Non-CKD** | **P value** |
| --- | --- | --- | --- |
| Numbers | 565 | 502 |  |
| Age (mean +/- SD) | 53.7 +/- 16.0 | 47.1 +/- 21.7 | <0.001 |
| Sex (male, %) | 416 (73.6%) | 342 (68.1%) | 0.056 |
| Ethnicity (Han, %) | 538 (95.2%) | 464 (92.4%) | 0.076 |
| Peak AEC (median [IQR], x10^9/L) | 0.77 (0.64-1.01) | 0.80 (0.66-1.05) | 0.160 |
| Study-specific AEC band |  |  | 0.492 |
| >0.52 to <1.00 (n, %) | 418 (74.0%) | 356 (70.9%) |  |
| 1.00 to <1.50 (n, %) | 92 (16.3%) | 88 (17.5%) |  |
| >=1.50 (n, %) | 55 (9.7%) | 58 (11.6%) |  |

**Supplementary Table 6. Multivariable logistic regression for persistent versus transient classification.**

| **Model** | **Predictor** | **Adjusted OR** | **95% CI** | **P value** |
| --- | --- | --- | --- | --- |
| Model 1: demographics | Age, per 10 years | 1.091 | 1.073-1.110 | <0.001 |
| Model 1: demographics | Male sex | 1.217 | 1.126-1.317 | <0.001 |
| Model 1: demographics | Han ethnicity | 1.463 | 1.260-1.699 | <0.001 |
| Model 2: demographics plus peak AEC | Age, per 10 years | 1.086 | 1.067-1.104 | <0.001 |
| Model 2: demographics plus peak AEC | Male sex | 1.211 | 1.118-1.312 | <0.001 |
| Model 2: demographics plus peak AEC | Han ethnicity | 1.527 | 1.310-1.781 | <0.001 |
| Model 2: demographics plus peak AEC | Peak AEC, per doubling | 1.754 | 1.672-1.841 | <0.001 |

*ORs use HC3 robust standard errors. Peak AEC was log2-transformed, so its OR is per doubling.*

**Supplementary Table 7. Cohort screening process and classification criteria for transient and persistent eosinophilia.**

| **Screening stage or group** | **n** | **Definition or criterion** |
| --- | --- | --- |
| Candidates after exclusion of foreign patients and duplicate records | 38,842 | At least one AEC >0.52 x 10^9/L during 2024-2025 |
| Excluded before classification | 15,773 | Only one total AEC measurement or <30 days between the earliest and latest available AEC measurements |
| Included study cohort | 23,069 | >=2 total AEC measurements with an observed span >=30 days |
| Transient eosinophilia | 19,745 | Met the repeat-measurement criterion but did not meet the persistence criterion |
| Persistent eosinophilia | 3,324 | >=2 AEC values >=0.50 x 10^9/L separated by >=30 days |

*All included patients had at least two AEC measurements spanning >=30 days before classification.*

**Supplementary Table 8A. Multivariable logistic regression for recorded CKD among 1,067 patients without an established eosinophilia-related diagnosis.**

| **Predictor** | **Adjusted OR** | **95% CI** | **P value** |
| --- | --- | --- | --- |
| Age, per 10 years | 1.192 | 1.119-1.271 | <0.001 |
| Male sex | 1.270 | 0.969-1.664 | 0.084 |
| Han ethnicity | 1.345 | 0.791-2.286 | 0.273 |
| Peak AEC, per doubling | 0.816 | 0.671-0.991 | 0.041 |

*The outcome was recorded CKD status. Peak AEC was log2-transformed, so its OR is per doubling.*

**Supplementary Table 8B. Adjusted associations between CKD stage and peak AEC outcomes among 565 patients with CKD.**

| **Outcome model** | **Predictor** | **Effect** | **Estimate** | **95% CI** | **P value** |
| --- | --- | --- | --- | --- | --- |
| Log2 peak AEC | CKD stage, per one-stage increase | Peak AEC ratio | 1.032 | 1.005-1.060 | 0.020 |
| Log2 peak AEC | Age, per 10 years | Peak AEC ratio | 1.007 | 0.987-1.027 | 0.486 |
| Log2 peak AEC | Male sex | Peak AEC ratio | 1.037 | 0.967-1.112 | 0.306 |
| Log2 peak AEC | Han ethnicity | Peak AEC ratio | 0.997 | 0.852-1.166 | 0.969 |
| >=1.50 band | CKD stage, per one-stage increase | Odds ratio | 1.262 | 0.982-1.622 | 0.069 |
| >=1.50 band | Age, per 10 years | Odds ratio | 1.018 | 0.855-1.211 | 0.844 |
| >=1.50 band | Male sex | Odds ratio | 1.456 | 0.730-2.906 | 0.287 |
| >=1.50 band | Han ethnicity | Odds ratio | 0.890 | 0.245-3.227 | 0.859 |

*Models were adjusted for age, sex, and ethnicity. The peak AEC model used log2-transformed AEC.*
